# *Plasmodium vivax* MSP1-42 kD Variant Proteins Detected Naturally Induced IgG Antibodies in Patients Regardless of the Infecting Parasite Phenotype in Mesoamerica

**DOI:** 10.3390/life13030704

**Published:** 2023-03-06

**Authors:** Lilia Gonzalez-Ceron, Barbara Dema, Olga L. Palomeque-Culebro, Frida Santillan-Valenzuela, Alberto Montoya, Arturo Reyes-Sandoval

**Affiliations:** 1 Regional Centre of Public Health Research, National Institute for Public Health Research, Tapachula 30700, Mexico; 2 Pandemic Science Institute, Nuffield Department of Medicine, University of Oxford, Oxford OX3 7BN, UK; 3 Parasitology Department, National Centre for Diagnosis Reference, Ministry of Health, Managua 11165, Nicaragua; 4 Instituto Politécnico Nacional (IPN), Unidad Adolfo López Mateos, Av. Luis Enrique Erro s/n., Mexico City 07738, Mexico; 5 Centro de Investigación en Ciencia Aplicada y Tecnología Avanzada (CICATA), Unidad Morelos, Instituto Politécnico Nacional (IPN), Boulevard de la Tecnología, 1036 Z-1, P 2/2, Atlacholoaya 62790, Mexico

**Keywords:** *Plasmodium vivax*, merozoite surface antigen-1, PvMSP1_42_, recombinant proteins, natural antibody responses, IgG, infected individuals, native antigen of blood stages, Mexico, Nicaragua

## Abstract

Background: The serological tests using blood stage antigens might be helpful for detecting recent exposure to *Plasmodium* parasites, and seroepidemiological studies would aid in the elimination of malaria. This work produced recombinant proteins of PvMSP1_42_ variants and evaluated their capacity to detect IgG antibodies in symptomatic patients from Mesoamerica. Methods: Three variant *Pvmsp1_42_* genes were cloned in the pHL-sec plasmid, expressed in the Expi293F™ eukaryotic system, and the recombinant proteins were purified by affinity chromatography. Using an ELISA, 174 plasma or eluted samples from patients infected with different *P. vivax* haplotypes were evaluated against PvMSP1_42_ proteins and to a native blood stage antigen (NBSA). Results: The antibody IgG OD values toward PvMSP142 variants (v88, v21, and v274) were heterogeneous (*n* = 178; median = 0.84 IQR 0.28–1.64). The correlation of IgG levels among all proteins was very high (spearman’s rho = 0.96–0.98; *p* < 0.0001), but was lower between them and the NBSA (rho = 0.771; *p* < 0.0001). In only a few samples, higher reactivity to the homologous protein was evident. Patients with a past infection who were seropositive had higher IgG levels and lower parasitemia levels than those who did not (*p* < 0.0001). Conclusions: The PvMSP1_42_ variants were similarly efficient in detecting specific IgG antibodies in *P. vivax* patients from Mesoamerica, regardless of the infecting parasite’s haplotype, and might be good candidates for malaria surveillance and epidemiological studies in the region.

## 1. Introduction

Malaria is a disease caused by protozoan parasites of the genus *Plasmodium*, and *Plasmodium vivax* and *Plasmodium falciparum* stand out as the most prevalent species, which are transmitted by *Anopheline* mosquitoes. In the Americas, *P. vivax* causes high mobility, causing 71.5% of the 596 and 552 cases reported in 2021 [1]. In Mexico, the number of malaria cases has shown a downward trend in the last 20 years, and between 2015 and 2020, a reduction of about 25% of cases was shown. Mexico shares borders with Guatemala, and this country has not reported *P. falciparum* cases since 2017. However, the Mesoamerican region face instability in the transmission of malaria. Belize, Guatemala, and Honduras reported a reduction of ≥40% of malaria cases until 2019. However, in Honduras, the malaria cases increased in the years 2020–2021, and the *P. vivax* cases represented a higher proportion. Likewise, Nicaragua, Costa Rica, and Panama showed notable and constant increases in malaria cases caused mainly by *P. vivax* and *P. falciparum* (>40%) [1].

Many countries are integrating efforts towards the elimination of malaria; however, the high burden of asymptomatic infections, low parasitemia level, hypnozoite carriers, drug resistance, and limited knowledge of the insecticide–resistance vector, among other factors [2,3], require additional efforts toward detecting and monitoring the temporal and geographic distribution of the parasite burden. *P. vivax* is difficult to eliminate and persists in affected areas after *P. falciparum* has been eliminated, in part attributed to its higher diversity [4,5]. In the initial stage of the blood infection with this species, gametocytes are produced and can be transmitted by several mosquito species [6]. More exceptional is the lodging of hypnozoites in the liver, which causes relapse episodes after weeks, months, or years after the primary blood infection, and its pattern depends on the strain and geographic region [7]. To date, no treatment regimen with PQ is completely effective in eliminating *P. vivax* hypnozoites in all treated individuals [8].

Serological tools are highly sensitive for detecting most individuals recently exposed to the parasite, especially when the malaria cases are significantly reduced [1]. These tools might be capable of delineating clusters of infection, targeting treatments, monitoring the effect of anti-malarial interventions, and certifying the interruption of transmission, among other factors [2,9,10,11,12]. Malarial blood infection induces specific antibody responses that are short-lived and might be boosted by subsequent infections [13,14], and these responses can be detected using laboratory methods. The use of a native antigen from blood stages allowed the efficient detection of specific IgG antibodies in naturally infected patients [15]. These antibodies fade out after a few months, although only if the malaria infection is cured [13,14], suggesting that seropositivity might be linked to a persistent infection or a re-infection in patients from southern Mexico [14]. Previous studies have suggested that serological tools might be useful in predicting *P. vivax* transmission risks, as the annual seropositivity rate was related to the annual parasite rate in the following year [16].

Sustainable and reproducible immunoassays using immunogenic proteins of *P. vivax* have recently been used for seroepidemiological studies and surveillance, and to evaluate protective immunity [2,17,18]. *P. vivax* MSP1-42 kD (PvMSP1_42_) is the C-terminal fragment participating in the first contact of the reticulocyte prior merozoite invasion, and consists of two fragments of 33 kD (PvMSP1_33_) and 19 kD (PvMSP1_19_). In the Mesoamerican region, PvMSP1_33_ has a polymorphic segment and PvMSP1_19_ is highly conserved [19,20]. PvMSP1_19_ has been shown to be highly immunogenic and successful in monitoring the transmission of malaria in some countries [6,21]. A meta-analysis using data from cross-sectional studies revealed significant heterogeneity in the association of IgG responses to *Pv*MSP1_19_ and *P. vivax* infection [12], making it suitable as a serological marker of *P. vivax* exposure. This study also uncovered the lack of studies in other regions of the Americas outside Brazil. Furthermore, the evaluation of 300 *P. vivax* molecules identified PvMSP1_19_ as one of the eight most reactive proteins [22], with appropriate characteristics for seroepidemiological surveillance [23].

In this work, three recombinant PvMSP1_42_ proteins that are highly frequent in *P. vivax* variants from southern Mexico were produced in a highly efficient eukaryotic system, Expi293. They were used to evaluate their ability to detect specific IgG antibodies in plasma and eluted samples from symptomatic individuals diagnosed with *P. vivax* infection from Mesoamerica.

## 2. Materials and Methods

The Ethics Committees of the National Institute of Public Health of Mexico (CI1718) approved the study. The patients’ personal information was encrypted.

### 2.1. P. vivax Parasites

*P. vivax*-infected blood samples displaying different PvMSP1_42_ haplotypes from southern Mexico [20] were used to produce three recombinant proteins (Figure S1). These variant proteins mirror the most frequent and divergent haplotypes from different haplogroups (HgA, HgB, and HgD) previously reported for Southern Mexico, which have fluctuated over time [20]. The diagnosis of *P. vivax* infection was made in symptomatic patients via a microscopy examination of a thick blood smear stained with Giemsa, as reported previously [19,20].

### 2.2. PCR Amplification

Genomic DNA was extracted using a QIAamp DNA Blood Mini Kit (Qiagen, Valencia, CA, USA) using the manufacturer’s procedure. *Pvmsp1* gene sequences were amplified by nested PCR, using primers containing the restriction sites of Agle1 and Kpn1: Pvmsp142FAge1 5′ attaccggtGCCGAGGACTACGACAAAG 3′, Pvmsp142R1 5′ CAAGCTTAGGAAGCTGGAGG 3′, and Msp142R2Kpn1 5′ gataggtaccCTCAAAGAGTGGCTCAGA 3′. For the PCR1 and PCR2 reactions, 20 μL of Pvmsp142FAge1 and Pvmsp142R1 and 40 μL of Pvmsp142FAge1 and Msp142R2Kpn1 were prepared, respectively. AccuPrime™ *Pfx* DNA Polymerase (Thermo Fisher Scientific Inc., Waltham, MA, USA) samples at 0.5 μM and 1 μL of the template DNA were added (for PCR2, 2 μL of PCR1). The PCR conditions were similar for PCR1 and PCR2: denaturation at 95 °C for 5 min; 35 cycles: denaturation at 94 °C for 1 min, melting temperature 59 °C for 1 min and extension at 72 °C for 75 s. The final extension phase was at 72 °C for 10 min. Amplification reactions were run in the ProFlex™ PCR System (Invitrogen, Carlsbad, CA, USA). To verify that the PCR products were of the expected molecular size, samples were run in agarose gels at 1% using SYBR Safe (Invitrogen, Carlsbad, CA, USA) at 120 V for 30 min. The molecular marker 1 kb plus ladder (Invitrogen, Carlsbad, CA, USA) was loaded as reference.

### 2.3. Cloning of DNA Fragments in the Expression Vector and Production of Plasmids with Pvmsp1_42_ Variant Genes

The pHL-sec plasmid (Addgene https://www.addgene.org/ (accessed on 3 March 2023)) was used [24]. Target DNA samples were recovered from agarose gels and purified using a QIAquick^®^ Gel Extraction Kit (Qiagen, Valencia, CA, USA) following the manufacturer’s instructions. The DNA fragments and the expression vector pHLsec were treated with the restriction enzymes AgleI and kpnI (New England Biolabs, Ipswich, MA, USA). In addition, the vector DNA was treated with alkaline phosphate to delete any phosphate group and avoid self-ligation. After treatment, the DNA samples were resolved in 1% agarose gels and a single DNA band was cut out and purified using a gel purification kit (Qiagen, Valencia, CA, USA), as indicated above. Each variant gene was used to ligate to pHLsec vector in a 20uL reaction.

The plasmids were inserted in highly efficient NEB^®^ 5-alpha competent *Escherichia coli* following the manufacturer´s instructions (New England Biolabs, Ipswich, MA, USA). Different dilutions of the transformed bacteria were spread on LB agar plates prepared with carbenicillin at 100 ug/mL and incubated overnight at 37 °C. For each plasmid variant, 10 individual colonies were picked up; each colony was inoculated on LB agar medium with added carbenicillin and incubated overnight at 37 °C under shaking for 12 h. The DNA plasmids were purified using a Qiagen Plasmid Midi Kit DNA plasmid extraction kit or QIAGEN Plasmid Plus Mega kit (Qiagen, Valencia, CA, USA) following the manufacturer´s instructions. The purified plasmids were kept at −20 °C.

The selected plasmid clones were sequenced using forward and reverse primers of the vector, with 5 μL per reaction of plasmid DNA at 100 ng/μL and 3.2 pmol/μL of each primer/reaction. Primers PHLsec-F 50 µM Pcag-F 5′GCAACGTGCTGGTTATTGTG 3′; PHLsec-R 50 µM Pglob-Pa-R 5′AGAAAAAGGGAGACGGTTTT 3′. Amplified DNA samples were sequenced at Biosciences, Oxford, UK (www.sourcebioscience.com (accessed on 3 March 2023)) and it was verified that the sequences matched the expected proteins. 

### 2.4. High-Yield Protein Expression of PvMSP1_42_ Variants in Expi293 Eukaryotic System

Expi293F™ cells (Thermo Fisher Scientific Inc., Waltham, MA, USA) were thawed and growth in Expi293™ expression medium for 3 weeks prior to transfection, following the manufacturer’s protocols (Thermo Fisher Scientific Inc., Waltham, MA, USA). These cells expressed peptides of N-linked glycosylated. The asparagine (Asn) residues ranged 6.8–7.3% in the expressed haplotypes. The suspended cells were grown in an orbital incubator at 120 RPMI, 37 °C, 8% CO_2_, and 80% humidity (Minitron™ CO_2_ orbital shaker; Infors HT, Bottmingen, Switzerland) in plastic flasks with ventilated caps (Corning^®^ Erlenmeyer sterile polycarbonate with 0.2 μm ventilated caps), as indicated by the manufacturers. For transfection, Expi293 cells at the exponential growth rate were adjusted to 3 × 10^6^ cells/mL in 60 mL of expression medium. Here, 1 μg of plasmid DNA was used to transfect 1 mL of the above culture. The plasmid DNA samples and ExpiFectamine^TM^ were prepared in Opti-MEM following the manufacturer’s protocol (Thermo Fisher Scientific Inc., Waltham, MA, USA). The transfected cells were incubated for 16–20 h, and prior enhancers were added dropwise with gentle shaking. The transfected cultures (cell density and viability) were monitored for the optimal expression of proteins and the supernatants were harvested at days ~4–4.5 (optimal density and viability of 83–90%). After centrifugation of the cell cultures, the supernatants were filtered to 0.2 μm and frozen at −20 °C until achieving protein purification.

### 2.5. Affinity Purification of Recombinant Proteins from the Eukaryotic System and the Analysis of Their Characteristics

Protein purification was carried out using affinity chromatography (AC) in AKTA^TM^-start (GE Healthcare, Chicago, IL, USA) using 1 mL HiTrap TALON Crude columns (Merk KGaA, Darmstadt, Germany). Prior to the protein purification, cell culture supernatants were dialyzed 3× against 1× PBS pH 7.4 at 4 °C, and one TALON column per variant protein was used. The columns were washed with dH_2_O Milli-Q water. The program to run a sample was as follows: pre-column pressure of 0.5 Mpa, maximum delta column pressure of 0.3 Mpa, default flow rate of 0.8 mL/min, max flow rate of 4 mL/min, default linear flow rate of 155.91 cm/h, maximum linear flow rate of 623.63 cm/h, minimum pH value (short-term) of 2, maximum pH value (short-term) of 14, minimum pH value (long-term) of 4, minimum pH value (long-term) of 12. The run protocol was as follows: prime and equilibration method equilibration volume (10 column volumes), sample application, wash-out unbound (10 mL), and elution and fractionation. The last prime-and equilibration volume was of 5 mL. The sample flow was standard at a rate of 0.8 mL/min. The binding buffer contained 150 mM of NaCl, 10 mM of TRIS, and 5% glycerol, and for the elution 200 mM of imidazole was added to the previous buffer. About 10 fractions of ~700 µL were obtained. The protein concentration was measured in a NanoDrop™ 1000 Spectrophotometer (Marshall Scientific, Hampton, NH, USA). Polyacrylamide gel electrophoresis (PAGE) results and Western blots were used to examine the protein purification. The protein parameters were obtained at https://web.expasy.org/cgi-bin/protparam/protparam (accessed on 3 March 2023).

### 2.6. PAGE and Western Blot Analysis

Here, 5 to 12 µL of supernatants or 1 µL of purified protein was prepared in Laemmli sample buffer (Bio-Rad Inc., Walford, UK), with or without 2-mercaptoethanol at 10%, and incubated at 95 °C for 5 min. The Mini-PROTEAN^®^ TGX^TM^ pre-cast gels of the 5–20% gradient (Bio-Rad Inc., Watford, UK) and the molecular weight marker Precision Plus Protein™ Western™ standards (Bio-Rad Inc., Walford, UK). The samples were run in 1× Tris/Glycine/SDS buffer for 75 min at 120 volts in a Mini-PROTEAN^®^ Tetra Vertical Electrophoresis Cell (Bio-Rad Inc., Walford, UK), and the gels were stained with Coomasie blue. For the Western blots, proteins resolved in PAGE were transferred to PDF membranes and 25 mM Tris, 192 mM glycine, and 0.1% sodium dodecyl sulfate, using a Bio-Rad Trans-Blot^®^ Semi-Dry System (Bio-Rad Inc., Walford, UK). The membrane was blocked with 5% non-fat dried bovine milk (Sigma Aldrich, Saint Louis, MO, USA) in PBS 1× at pH 7.4 under shaking. Then, one blot was incubated with (a) mouse anti-6His mAb IgG (Invitrogen, Carlsbad, CA, USA), diluted at 1:2000 in 1× PBS −2.5% non-fat milk and 0.05% tween, and incubated overnight at 4 °C. After, the membranes were intensively washed 3 times. The goat anti-mouse IgG (H + L)-HRP (Bio-Rad) at 1:2000 was incubated for 1 h at RT. After intensive washing, the reactions were developed using the Clarity Western ECL substrate (Bio-Rad Inc., Walford, UK), and the images were recorded in ChemiDoc XRS+ (Bio-Rad Inc., Walford, UK) at different exposition times. Other blots were incubated with immune and non-immune serum malaria samples followed by an antibody anti-human IgG-HRP at 1:3000.

### 2.7. Samples to Test

One-hundred and fourteen plasma samples and 64 blood samples impregnated onto filter paper from *P. vivax* patients were obtained from two sites of the Mesoamerican region, in southern Mexico (2002–2009) and Nicaragua (2012–2013) [19,20], respectively. All samples were preserved at −80 °C until use. The PvMSP1_42_ phenotypes of the parasites from those infected individuals were previously published [19,20]. A group of 36 plasma samples from individuals with no history of *P. vivax* infection were also evaluated and used to estimate the cut-off value. From each filter paper dried blood sample, two circles of approximately 5 mm each were cut out and placed in an Eppendorf tube, and 250 µL of 1× PBS − 0.01% thimerosal—5% glycerol was added to obtain a dilution of ~1:50. The antibodies were eluted at 4 °C overnight, and the next day the samples were vortexed gently before the remaining filter papers were discarded. The plasma samples were also pre-diluted to 1:50 in PBS–thimerosal–glycerol.

### 2.8. ELISA Tests

A crude extract of the blood stages (NBSA) was previously prepared and preserved in liquid nitrogen. Plasma samples and eluates from the filter paper were tested at a 1:500 dilution, and the ELISA procedure was similar to that reported previously [14]. To monitor the inter-plate consistency, a previously prepared pool of immune plasma and a pool of non-immune plasma were used as the controls, at 1:2000 and 1:500 dilutions, respectively [14].

Other ELISAs were standardized to detect IgG antibodies against three recombinant proteins as follows. Flat-bottom 96-well Maxisorb plates were coated with 50 µL of different PvMSP1_42_ proteins (v88 or v21 or v274) diluted in 1× PBS pH 7.4 at 1 µg/mL and incubated overnight at room temperature (~25 °C). After the unbound protein was removed, the wells were blocked using 5% non-fat dried milk in 1× PBS for 1 h. The plasma samples or eluates of filter paper at 1:1000 dilution and controls in a pool of immune plasma samples at 1:4000 and non-immune plasma samples at 1:1000 (similar to the tested samples) were diluted in 2.5% non-fat milk and 0.05% Tween in 1× PBS, added in duplicate, and incubated for 2h at R.T. The plates were washed 3 times with 0.05% Tween in 1× PBS, then a goat anti-human IgG–HRP (Merck, KGaA, Darmstadt, Germany) solution diluted to 1:3000 in a diluent buffer was added and incubated for 1 h. Once again, the plates were washed as before. The reaction was developed using a TMB ultrasensitive substrate (Merck, KGaA, Darmstadt, Alemania) and incubated for 20 min. The reaction was read at 630 in a spectrophotometer (Agilent, Santa Clara, CA, USA). The OD values of the reagents (or PBS) were consistently below 0.05 for recombinant proteins using the TMB substrate, whereas it was ~0.3 for the NBSA using ABTS as the substrate. The average optical density (OD) of the duplicates was calculated per sample and the OD values from the PBS (per plate) were subtracted. The ELISAs were run blinded, as the specialized technician who ran the tests had no access to the sample information.

### 2.9. Data Analysis

The statistical analysis results and graphs were prepared in Stata v12.1 (StataCorp LLC Lakeway, College Station, TA, USA) and R v4.2.1 (https://www.r-project.org/ (accessed on 15 August 2022)), respectively. The cut-off values were defined using the samples from non-immune patients as the mean +3 SD for each type of coating antigen. Differences in proportions were evaluated using the chi-square (χ^2^) test. Differences in numeric variables were calculated using two-sample Wilcoxon rank-sum (Mann–Whitney) test or Kruskal–Wallis equality-of-populations rank test. The linear correlations between numeric variables, such as IgG OD values from recombinant proteins versus the NBSA and those versus age, parasitemia, and the number of days with symptoms, were determined using Spearman´s rho correlation coefficient. The correlation scale 0–0.19 “very weak”, 0.20–0.39 “weak”, 0.40–0.59 “moderate”, 0.60–0.79 “strong”, and 0.80–1.0 “very strong” was used as previously suggested (https://www.statstutor.ac.uk/resources/uploaded/spearmans.pdf (accessed on 3 March 2023)).

## 3. Results

### 3.1. Recombinant Protein Purification and Reactivity as Assessed by Western Blot

**Figure 1:**
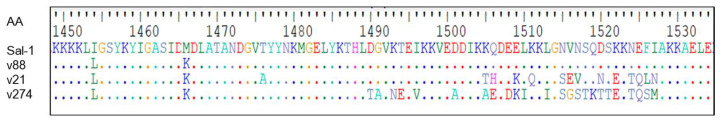
Amino acid polymorphism of PvMSP1-42kD protein variants (v88, v21, and v274) comprising amino acid residues 1328–1724 (397 residues), using Sal-1 as the reference (XM_001614792). Amino acids are indicated by one letter code. The image was obtained from Bio Edit v7.2 (https://bioedit.software.informer.com/, accessed on 15 January 2023) and shows the main polymorphic segment. Two other amino acid changes were observed in all proteins: at 1347 residues G → W, and at 1361 residues A → E. Protein variant v88 was the most similar to the Sal-1 sequence. AA, amino acid position.

Three PvMSP1_42_ variant proteins were successfully obtained: labelled v88, v274, and v21, corresponding to haplotypes h8, h1 and h2, respectively (Figure S1). These proteins were consistently obtained at high concentrations from 60 mL of culture supernatant at about 200–790 µg/mL in fractions f4 and f5. The protein variant v88 was obtained at a higher concentration than the other proteins. The amino acid polymorphism among the recombinant proteins is shown in Figure 1, and also Asn residues were observed; HgA (v88) similar to sal1 had Asn residues at positions 1515, 1517, and 1524; in HgB (v21), the residues were at different positions 1520 and 1527, with one residue at position 1493 in HgD (v274).

**Figure 2:**
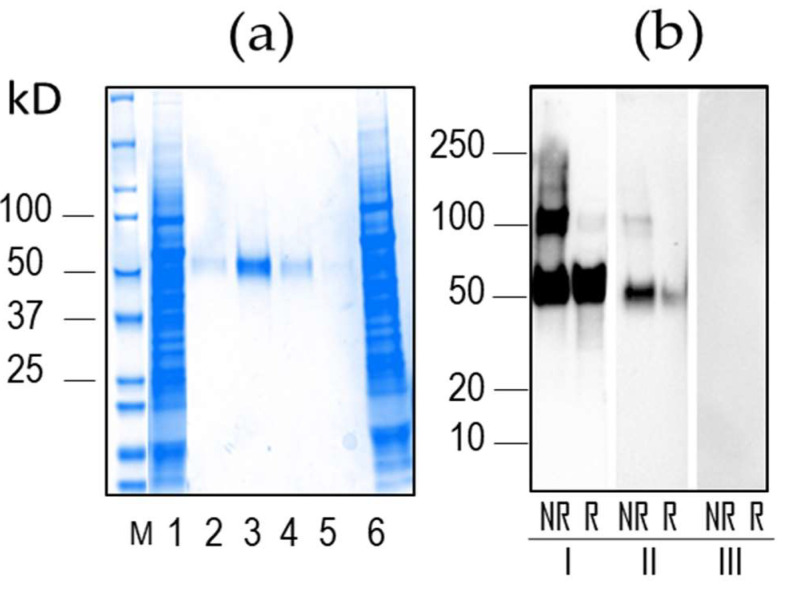
*P. vivax* MSP_42_ v88 protein purification and Western blot analysis. (**a**) PAGE 5–20% analysis using 1 µL of proteins purified by affinity chromatography fractions (f): f3, f4, f5, and f6 (lanes 2–5, respectively); culture supernatant (lane 1, 12 µL); flow-through (lane 6, 12 µL). (**b**) Purified proteins were run in PAGE gradient gels at 5–20% and transferred to PVD membranes. Blots were incubated with (I) mouse mAb IgG anti-histidine, followed by anti-mouse-HRP (5 s exposition), (II) *P. vivax* immune serum (20 s exposition), and (III) non-malaria immune serum (20 s exposition), followed by an anti-human IgG-HRP. Blots were developed using chemiluminescence. NR, non-reducing; R, reducing conditions. M, molecular marker.

The histidine-tagged proteins comprised 437 amino acids (including the signal peptide), with a molecular weight of 49.5 kD and a theoretical isoelectric point of 5.46. The recombinant proteins were successfully purified by AC, showing a single band of ~50kDa on PAGE gels stained with Coomassie blue (Figure 2) and reacted with an anti-histidine mAb using Western blot assays (Figure 2). Specific reactivity with *P. vivax* immune plasma was also evident for proteins (e.g., v88) under reducing and non-reducing conditions. The reaction was most intense when the proteins were used under non-reducing conditions; this high reactivity made the proteins good candidates for immunoassays (ELISA).

### 3.2. IgG Antibody Levels to PvMSP1_42_ Proteins and the NBSA as Assessed by ELISA

The IgG OD values toward the NBSA and toward PvMSP1_42_ recombinant proteins v88, v21, and v274 were obtained for 114 plasma samples and 64 eluted samples from infected patients. The ELISA reads obtained at 20 min after adding the stop solution yielded highly polarized OD values (Figure S2) and high background noise (non-immune samples, mean + 3 SD ~0.5). Therefore, we preferred to use a low cut-off value for the TMB substrate and OD readings at 630 NM. A comparison of IgG OD values of (1) samples from patients, (2) non-immune individuals, and (3) pools of immune and non-immune controls run in different plates is shown in Figure S1. Due to the high consistency plate-to-plate of the OD values of the controls, the row data were not normalized to avoid erroneous adjustments.

The IgG OD levels were heterogeneous against all PvMSP1_42_ proteins (OD_630_) and to the NBSA (OD_405_). Table 1 shows comparisons of IgG OD values and seropositivity rates using PvMSP1_42_ proteins and the NBSA. Among the recombinant proteins, no differences in IgG OD values were observed (n = 178, *X*^2^ = 1.48, d.f. = 2, *p* = 0.477).

**Figure 3:**
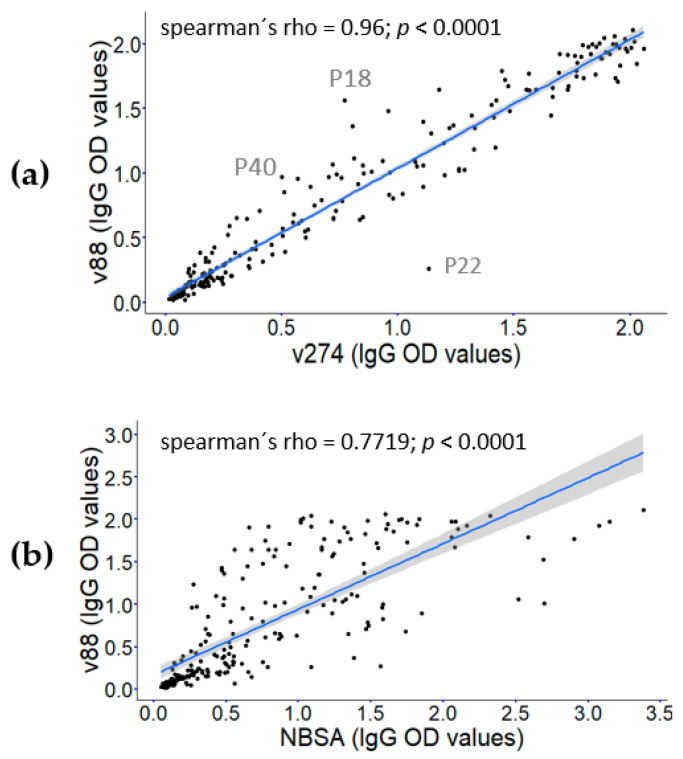
Correlation between IgG OD values for *P. vivax* MSP1_42_ proteins and the NBSA. (**a**) Correlation between IgG OD values for MSP1_42_ proteins v88 and v274. Sample from patient P22 yielded a four-fold higher IgG OD value for the homologous protein v274 than for the heterologous v274. Samples P18 and P40 yielded two-fold higher IgG OD values for the homologous protein v88 than the heterologous ones v274 and v21. (**b**) Correlation between IgG OD values for the NBSA and MSP1_42_ v88 protein. One-hundred and seventy-eight plasma and eluted samples were included.

Using 178 samples from *P. vivax*-infected patients, a very strong correlation was detected when comparing the IgG OD values for PvMSP1_42_ variants v88 (Figure 3a), v274, and v21 (*n* = 178, spearman coefficient rho = 0.96–0.98, *p* < 0.0001). Likewise, a strong correlation was observed between the IgG OD values for the three PvMSP1_42_ proteins (v88, v21, v274) and for the NBSA (rho = 0.77–0.78, *p* < 0.0001) (Figure 3b). If the IgG OD values were disaggregated by country (plasma vs. eluted samples), the correlation coefficients of IgG OD values remained similar among the recombinant proteins and between them and the NBSA. For Nicaraguan samples, IgG OD values: among recombinant proteins (rho = 0.722–0.741; *p* < 0.0001) or between them and the NBSA (rho = 0.98–0.99; *p* < 0.0001). For plasma samples (southern Mexico), IgG OD values: among recombinant proteins (rho = 0.971–0.979; *p* < 0.0001), or between them and the NBSA (rho = 0.772–0.805; *p* < 0.0001).

In only a few samples the IgG OD values were affected by heterologous PvMSP142 proteins, producing lower OD values (Figure 3a), e.g., sample P22 yielded a four-fold higher IgG OD value with the homologous protein v274 (representing haplogroup HgD; OD value 1.132) than with heterologous v88 (HgA) or v21 (HgB), with OD values of 0.217 and 0.259, respectively. This sample was seropositive to the NBSA with an IgG OD value of 1.085. The samples from patients P18 and P40 yielded a two-fold higher IgG OD values with homologous protein v88 (the OD values for samples P18 and P40 were 1.565 and 0.972, respectively) than to the heterologous ones v274 and v21 (OD value for P18 of 0.768 (both proteins) and for P40 of 0.499/0.67, respectively), and were seropositive (OD values of 0.831 and 0.297, respectively) to the NBSA. Some samples with low IgG OD values for homologous haplotypes were seronegative to heterologous ones, as described in 3.4.

IgG OD values for plasma versus eluted samples to PvMSP1_42_ v21 or v274 were not different (z = 1.158, *p* = 0.24; z = 0.382, *p* = 0.70, respectively). Only for PvMSP1_42_ v88, IgG OD values were higher for plasma (Mexican) than eluted samples (Nicaragua) (z = 2.33; *p* = 0.019), and the difference in IgG OD values for the NBSA was marginal (z = 2.025; *p* = 0.043).

### 3.3. IgG Antibodies to MSP142 Proteins and Infecting Parasite’s Haplotype

*Pvmsp1_42_* sequences from the infecting parasites of the patients were used to prepare a haplotype network as previously described [20,25], and the genetic relationships of the parasites based on the *pvmsp1_42_* gene are shown in Figure S3. The plasma and eluted samples were grouped by their parasite´s haplotype found in the infected host.

**Figure 4:**
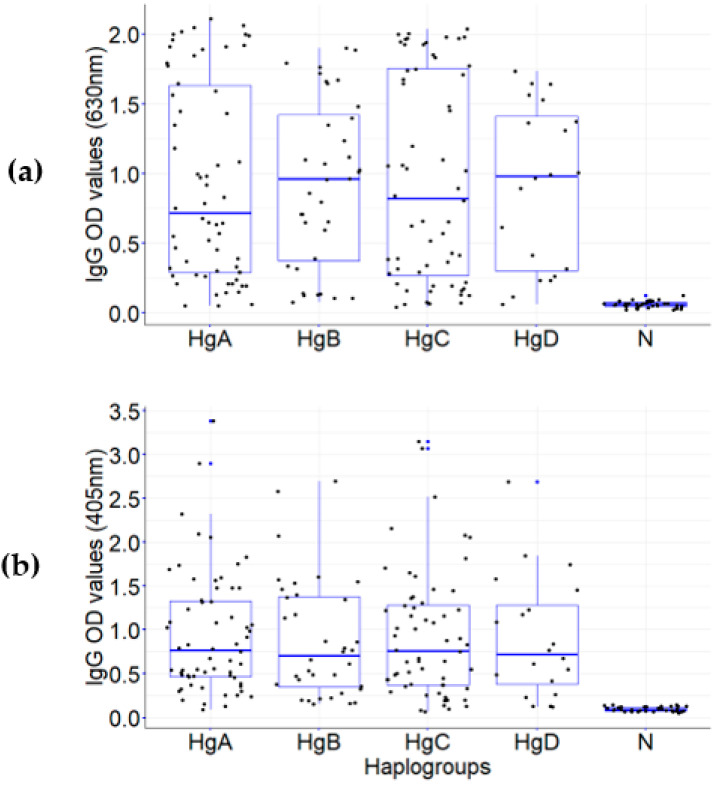
IgG OD values against *P. vivax* in plasma and eluted samples from symptomatic individuals infected with different parasite haplotypes: (**a**) PvMSP1_42_ v88 protein; (**b**) NBSA. Samples were grouped by the *P. vivax* haplotype (HgA, B, C, D) causing the acute infection. Group N corresponds to samples from individuals without previous malaria infection. Kruskal–Wallis equality-of-populations rank test: IgG OD_405_ to MSP1_42_ v88 and to the NBSA among four haplogroups, *X*^2^ = 0.132, d.f. = 3, *p* = 0.98, and *X*^2^ = 0.360, 3 d.f., *p* = 0.948, respectively, among those groups and the non-immune group to PvMSP1_42_ v88 (*X*^2^ = 80.08, d.f. = 4, *p* = 0.0001) and to the NBSA *(X*^2^ = 82.78, d.f. = 4, *p* = 0.0001). HgA, *n* = 62; HgC, *n* = 36; HgB, *n* = 60; 5; HgD, *n* = 20. N, non-immune samples (*n* = 36).

No differences were detected between the IgG OD values of samples from patients infected with different haplogroups (HgA, B, C, D) (e.g., for PvMSP1_42_ v88: *X*^2^ = 0.132, d.f. = 3, *p* = 0.98), similar to variants PvMSP1_42_ v21 and v274. Likewise, no differences were observed for IgG OD values for the NBSA among haplogroups: *X*^2^ = 0.360, d.f. = 3, *p* = 0.948 (Figure 4). However, a difference between IgG OD values for PvMSP1_42_ proteins (e.g., v88) and the NBSA between the haplogroups and the non-immune group (N) was evident (*X*^2^ = 80.08, d.f. = 4, *p* = 0.0001; *X*^2^ = 82.78, d.f. = 4, *p* = 0.0001, respectively) (Figure 4).

### 3.4. Discordances between IgG Antibodies to PvMSP1_42_ Recombinant Proteins (v88, v21, and v274) and to the NBSA

One-hundred and sixty-two samples (91%) had IgG Od values above the cut-off value for at least one recombinant protein (OD values >0.15), at 87.6% for v88, 84.8% for v21, and 85.4% for v274 (Table 1). The sample from patient P87 (haplotype h8 (v88)) produced an OD value of 0.261 for the homologous protein v88, while being seronegative for the heterologous proteins v21 and v274 (OD 0.09). This sample had an IgG OD value of 0.785 for the NBSA, whereas the sample from patient P136 (h8) had an IgG OD value of 0.152 for the heterologous PvMSP1_42_ v274 and negative borderline IgG OD values for PvMSP1_42_ v21 and for the homologous protein (v88) (0.149 and 0.146, respectively), while a positive IgG OD value of 0.674 for the NBSA was obtained.

Eighteen samples had IgG OD values for PvMSP1_42_ below the cut-off value (all proteins, OD values <0.15), from which 11 were also negative to the NBSA (OD values <0.2), and most of the other seven had borderline or low OD values (mostly <0.3, and only one sample had 0.56 OD value). Likewise, 17 samples with IgG OD values for NBSA <0.2 (seronegative) had IgG OD values for PvMSP1_42_ above the cut-off value (>0.15) though values were borderline or low, 5 for v88 (29.4%; OD values 0.23–0.34), 2 for v21 (11.7%; OD values 0.224 and 0.229), and 3 for v274 (17.6%; OD values 0.168–0.184).

Discordances between IgG OD values for PvMSP1_42_ proteins and to the NBSA were expected due to the difference of using a purified protein versus a multi-antigenic crude extract such as the NBSA, and because the magnitudes of antibody responses to different antigens vary among patients [18,26,27]. Additionally, the differences might also be influenced by the sensitivity of the substrates; TMB detects 20 pg/mL versus ABTS at 250 pg/mL, and the sample dilution used in the ELISA.

### 3.5. Relationships between IgG Antibodies to MSP1_42_ Proteins and to the NBSA with Age, Gender, Parasitemia, Days of Symptoms, and Previous P. vivax Infection

The individuals from southern Mexico were older than those from Nicaragua (women: *n* = 48, median 32 IQR 21.5–47 versus *n* = 33, median 19 IQR 13–29, respectively; z = 4.02, *p* = 0.0001; men: *n* = 66, median 31 IQR 18–44 versus *n* = 26, median 17 IQR 10–22, respectively; z = 3.39, *p* = 0.0007). The IgG OD values for PvMSP1_42_ v88 in samples from Nicaragua had no correlation with patient age (*n* = 59, Spearman´s rho = 0.189, *p* = 0.15). However, the IgG OD values for the NBSA showed a weak and positive correlation with age (Spearman’s rho = 0.269, *p* = 0.037). On the contrary, the IgG OD values for both MSP1_42_ v88 and the NBSA from Mexican samples correlated weakly and negatively with age only in men (*n* = 66, rho = −0.299, *p* = 0.015 and rho = −0.291 *p* = 0.018, respectively).

In addition, the samples from Mexico had information on the parasitemia, previous malaria infection, and the days with symptoms (which was calculated as the difference between the dates of blood collection and symptoms onset). Of the 114 *P. vivax*-infected individuals, the IgG OD values for MSP1_42_ v88 or the NBSA correlated weakly and negatively with the parasite density in the bloodstream (Spearman’s rho = −0.1839, *p* = 0.050 and rho = −206, *p* = 0.027, respectively). No correlation between the IgG OD values for MSP1_42_ v88 nor the NBSA and the days with symptoms was detected (Spearman’s rho = 0.081, *p* = 0.397 and rho = −0.086, *p* = 0.363, respectively).

**Figure 5:**
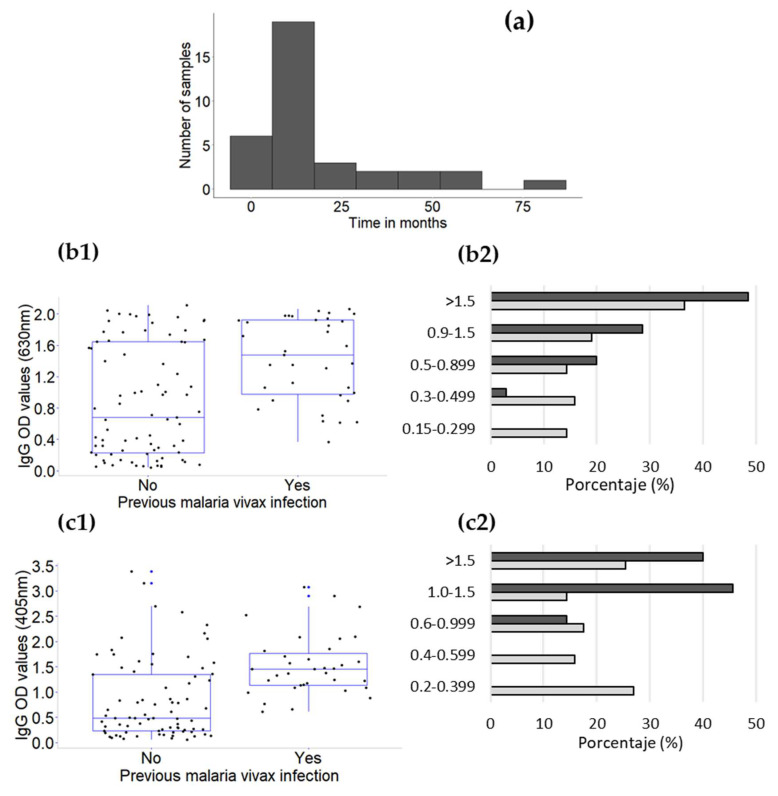
Comparison of IgG OD values among patients with and without past *P. vivax* infection from southern Mexico. (**a**) Distribution of samples from patients based on the number of months from the previous *P. vivax* infection (*n* = 35). IgG OD values for (**b**) PvMSP1_42_ v88 protein and (**c**) NBSA. (**b1**,**c1**) Comparison of IgG OD values of patients with and without (*n* = 79) past *P. vivax* infection to PvMSP1_42_ v88 protein (z= −3.88; *p* < 0.0001) and against NBSA (z= −4.73; *p* < 0.0001), respectively. (**b2**,**c2**) Distribution of IgG OD values (from very low to very high values) from samples of patients with (dark grey) or without (light grey) past infection.

Thirty-five of these patients (30.7%) had at least one previous *P. vivax* infection (median of 12 months, range from 3 to 84 months). The IgG OD values for the PvMSP1_42_ proteins were higher in this group than in patients suffering from a primo-infection (e.g., against MSP1_42_ v88, median OD value of 1.48 (range 0.365–2.06) versus median OD value of 0.678 (range 0.042–2.11), respectively; z = −4.26, *p* < 0.0001) (Figure 5). Likewise, the IgG OD values for the NBSA in patients with a previous infection were higher (median OD value of 1.45, range 0.611–3.075) than those in patients who had a primo-infection (median OD value of 0.487, range 0.062–3.381; z= −4.73, *p* < 0.0001). No relationship was detected between the time interval in months of previous infection and IgG OD values for PvMSP1_42_ v88 (Spearman’s rho = 0.277, *p* = 0.106) nor the NBSA (Spearman’s rho = 0.126, *p* = 0.472).

In fact, all individuals with reports of previous *P. vivax* infection were seropositive for IgG antibodies to PvMSP1_42_ proteins and to the NBSA; however, the OD values did not correlate (v88 vs. NBSA; Spearman’s rho = 0.2527, *p* = 0.1431). Considering only patients with a *P. vivax* primo-infection, the seropositivity rates were reduced to 71–76% and 81.6% to PvMSP1_42_ and NBSA, respectively. In addition, the IgG OD values above the cut-off value were grouped arbitrarily by their magnitude into very low (0.15–0.299), low (0.3–0.499), intermediate (0.5–0.899) high (0.9–1.499), and very high (>1.5) for PvMSP1_42,_ and using a similar scale for the NBSA (Figure 5(b2,c2), respectively). Patients with past infection had OD values that were mostly intermediate to very high, whereas 30.2% and 42.85% of those with a primo-infection had low or very low values (<0.5 or <0.6 OD) for PvMSP1_42_ v88 and the NBSA, respectively.

In addition, patients with a primo-infection had a higher parasitemia level (median = 9020 parasites/µL, range 284–59,600) than patients with a history of infection (median = 5680 parasites/µL, range 720–47,600; z = 2.368, *p* = 0.018). A weak and negative correlation was detected between parasitemia and IgG OD values for PvMSP1_42_ proteins, e.g., v88 (*n* = 63, rho= −0.318, *p* = 0.011), in patients with primo-infection and seropositive for at least one PvMSP1_42_ variant. In this regard, no correlation was found in patients with a past infection (*n* = 35, rho = −0.145, *p* = 0.403).

Regarding the days with symptoms before sample collection, more days with symptoms were registered for patients with a primo-infection (*n* = 79, median = 5 days, range 0–21) than those who had a past infection (median = 4 days, range 1–14; z = 3.097, *p* = 0.0020). Furthermore, a difference in the days with symptoms between individuals with a primo-infection and seropositive to at least one PvMSP1_42_ protein (*n* = 63, median 6, range 2–21) and individuals negative to all PvMSP1_42_ proteins (*n* = 16, median 4, range 0–8) was detected in patients with a primo-infection (z= −2.18, *p* = 0.029).

## 4. Discussion

Serological tools designed to detect individuals or areas with recent malaria infection would accelerate the elimination of malaria, essentially in areas with residual transmission. This would guide vector control processes and the implementation of treatment strategies. Current diagnostic tests, such as the microscopy of thick blood smears or rapid tests, have limitations when dealing with very low parasitemia levels in patients suffering from acute or chronic infection, or if the parasite densities fluctuate during infection. Recent evidence suggests a parasite reservoir in the spleen, which might be of a greater magnitude than that present in the bloodstream, probably facilitated by the high density of reticulocytes in this organ and the parasite proteins that mediate adherence [28,29]. The detection of IgG antibodies by the appropriate antigen could suggest recent infection even in asymptomatic patients, as has been reported using a native antigen of blood stages [14,30]. In addition, in individuals with a recent *P. vivax* infection, the presence of specific antibodies could indicate the possibility of posterior relapse episodes [14,31,32].

In this study, antibody responses to three divergent PvMSP1_42_ proteins from different haplotypes that were successfully produced in a highly efficient Expi293 system were highly efficient in detecting a specific IgG antibody in symptomatic patients from the Mesoamerican region (southern Mexico and Nicaragua). The range of *P. vivax* patients seropositive for PvMSP1_42_ proteins was 85–88%, slightly lower than for the native extract of blood stages, the NBSA (90.5%). However, the PvMSP1_42_ v88 haplotype, which is closely related to the Sal I strain and was detected highly frequent in southern Mexico [20] and Nicaragua [19], had a higher median IgG OD value than the v21 and v274 variants or the NBSA. The heterogeneity in the IgG OD values for PvMSP1_42_ proteins was similar to that observed for the NBSA, which was irrespective of the parasite haplotype. Heterogeneity in IgG responses against PvMSP1 fragments and other molecules has been observed in previous studies [27,33,34,35,36,37,38,39].

A meta-analysis revealed significant heterogeneity in the cross-sectional studies on the association of IgG responses against PvMSP-1_19_ with patent *P. vivax* infection (*I*^2^  = 73.8%, *p* = 0.004), highlighting the importance of comprehending antibody responses to *P. vivax* parasites in different regions, since most studies were carried out in Brazil and other regions of southeast Asia [12]. PvMSP1_19_ was found to be one of 8 proteins to have reported with recent *P. vivax* exposure (1–12 months after *P. vivax* infection) in low-transmission settings in Brazil, Thailand, and the Solomon islands [17,22,27,32], and is suggested to have about 80% sensitivity and specificity in detecting recent infections in low endemic regions [27]. Proteins produced in different expression systems have been used with good results, and the seropositivity rates vary among geographic areas. Using PvMSP1_19_ expressed in *E. coli*, 92.5% of the subjects from Brazil and suffering acute infection were IgG-seropositive [33]. Another study, using the protein expressed in non-glycosylated yeast protein and bacteria, detected 85.6% and 80.7% of the seropositive patients, respectively, from a low endemic region in Brazil [34]. This protein, which is expressed in *Saccharomyces cerevisiae,* detected IgG antibodies in 85% of infected patients from Turkey [35]. In an area with no recent transmission in Vanuatu, 12% of the samples were seropositve to PvMSP1_19_, and this study showed similar seropositivity rates to PfMSP1_19_ and to a schizont extract of 12.6% and 15%, respectively [40]. However, antibody levelswere not shown to explain the magnitude of the residual IgG responses.

Notwithstanding, a larger fragment such as PvMSP1_42_ might be more sensitive in detecting antibody responses. In Korean patients, the prevalence rate of IgG antibodies to PvMSP1_19_ was 89.6%, with rates of 48–64% to two variants of PvMSP1_33_ [36]. Wickramarachchi [41], using proteins expressed in vaculovirus, detected higher positivity rates using PvMSP1_42_ (71–77%) than PvMSP1_19_ (57–63%) in *P. vivax*-infected patients from a low endemic region in Sri Lanka. In Iran, 86.9% of patients with a *P. vivax* infection were ELISA-seropositive against PvMSP1_42_ expressed in *E. coli* [37]. The polymorphism of the PvMSP1_33_ segment mainly comprised 37 amino acids (1490–1527 amino acid position) in *P. vivax* from Mesoamerica [19,20], and seemed not to affect the detection of specific IgGs. In fact, very high correlation rates (98–99%) between protein variants were marked and little differences were noted in antibody levels among infecting parasite haplotypes, confirming the presence of conserved immunogenic B cell epitopes in PvMSP1_19_ [6,42,43], and probably in PvMSP1_33_ [20,33,36], which in most cases overcome the polymorphism at the PvMSP1_33_ segment, at least in this study. Conserved segments along the PvMSP1 protein are likely present, as the detection of antibodies to different homologous proteins was consistently accompanied by a reaction to unrelated variants [33]. Similar to our results, in Korean patients only a few samples (1.4%) were discordant for PvMSP1_33_ SalI versus Belem haplotypes [36].

Although Berhe et al. [44] suggested that MSP1 was not glycosylated, a more recent analysis indicated unusual N-glycosylation restricted to one or two GlcNAc residues in *P. falciparum* surface proteins [45,46]. No significant difference in IgG titers to PvMSP1_19_ expressed in either *E. coli* versus *S. cerevisiare* were detected in *P. vivax* patients in Brazil [47]. In our study, the N-glycosylation by Expi293F™ cells might have had no great effect on the antibody binding to the PvMSP1_42_ proteins because the high seropositivity was accompanied by a wide range of IgG OD values in samples from Mesoamerican patients, especially when using variant v88, a recombinant having more Asn residues in the polymorphic segment and higher IgG OD values than other variants.

The characteristics of the transmission of malaria, the populations at risk, and the transmission intensity might determine the magnitude and longevity of antibody responses, whereby in high transmission areas patients might accumulate parasite exposure and natural immunity, yielding long-lived antibody responses [26,27,48]. After repeated exposure, the antibody responses increase with age [49,50]. In our study, the IgG responses to PvMSP1_42_ showed a poor correlation with age, in agreement with the low malaria transmission rates taking place in Mesoamerica and because populations of different age groups are at risk. Only a weak positive correlation was observed between age and IgG responses to the NBSA in Nicaraguan patients. In this country, malaria infection was found to be distributed across all age groups [51]. Similarly, in low endemic regions of Brazil, no correlation of antibody responses and age was encountered [33].

In southern Mexico, some interesting differences were detected between IgG OD values between patients with a primo-infection and who had suffered a past *P. vivax* infection. Most patients with a past infection were seropositive with mid–high antibody responses to all PvMSP1_42_ variants and to the native antigenic NBSA. They might have boosted their antibody responses earlier, as these patients had less days with symptoms than those with the primo-infection. Nevertheless, 71–78% of patients with a primo-infection were seropositive to any ELISA antigen; furthermore, a proportion of them had low OD values, which could limit the usefulness of serological tests. Patients with a primo-infection and seronegative to all PvMSP1_42_ proteins might have had not yet generated enough IgG titers, as they had recorded less days with symptoms (0–8 days) than seropositive patients (2–21) at the time of sample collection. Others reported that a high proportion of patients seroconverted one week after symptom onset [13]. At day 7 after *P. vivax* diagnosis and using the NBSA antigen, 98.7% of *P. vivax*-infected patients from southern Mexico were seropositive [14]. Furthermore, the lack of correlation between antibodies to PvMSP1_42_ variants and the NBSA in patients with a past infection might be explained by the heterogeneity of antibody responses to different blood stage antigens by patients with a past infection [18,22,27,52]. Moreover, the higher parasitemia level detected in patients from southern Mexico with a primo-infection and lower IgG OD values than those with a past infection might correspond in part to the protective immune responses induced by PvMSP1_42_ [53] and the partial clinical protection observed earlier against PvMSP1_19_ [54].

The power of the serological test has been exposed, and combined with radical treatments might cause significantly reduced (59–69%) parasite prevalence [32]. It is known that antibody responses in treated patients fade out rapidly [13,14,55]; however, untreated or partially treated patients might develop chronic infections or relapse episodes. Our previous study showed that IgG antibodies to the NBSA fade out after several months after treatment, and in patients with a new blood infection, a boost in IgG antibodies was observed [14]. The fact that *P. vivax* patients from southern Mexico and Nicaragua showed a wide magnitude of antibody responses suggests these proteins as candidates for seroepidemiological studies and surveillance in Mesoamerica. However, in terms of elimination, a better understanding is required of the dynamics of antibody responses; seropositivity, the magnitude of IgG responses, and the longevity of IgG antibodies in follow-up patients with or without past infections from this region. In addition, the study of human populations from areas free of or with very low rates of malaria transmission is necessary to evidence the characteristics of residual antibodies and endorse the utility of serological tests in detecting patches of recent transmission, among other factors.

## 5. Conclusions

In this study, variants of *P. vivax* MSP1_42_ were produced in a highly efficient system, Expi293, which identified proteins exposing B cell epitopes and detected a wide range of IgG responses in *P. vivax* patients from Mesoamerica. The polymorphism of the PvMSP1_42_ is restricted to a small fragment, and generally seems not to affect the detection of IgG by heterologous haplotypes. In fact, a very strong correlation of IgG OD values for protein variants was observed among samples from *P. vivax* patients. All acute patients with a previous malaria infection were seropositive, and most had medium or high antibody titers. In contrast, only 71.79% of those with a primo-infection were seropositive, and from them, a proportion had very low or low IgG OD values, which might limit the utility of the serological tests and the interpretation of results if no other data from patients or the study populations are available. Further studies are necessary to evaluate the effect of N-glycosylation by Expi293F™ cells in antibody detection, and elucidate the IgG antibody dynamics in patients with different intensity levels of exposure to the parasite. Additionally, an evaluation of these proteins in the detection of specific IgG antibodies in malaria-free areas and regions with no recent transmission, and their ability to detect subtle changes in transmission would be a tremendous contribution to accelerating the elimination of malaria in the region.

## Figures and Tables

**Table 1:** IgG OD values of plasma and eluted samples from *P. vivax* symptomatic patients, measured using an ELISA against three PvMSP1_42_ protein variants and the NBSA.

Protein/Antigen	OD Values:					Type of Sample:	
	Median	IQR	Max	Min	Overall Positivity:	Plasma *n* = 114	Filter Paper *n* = 64
infected patients:					≥0.15		
MSP1_42_ v88 ^2^	0.848	0.289–1.647	2.11	0.042	156 (87.7%)	85.9%	90.6%
MSP1_42_ v21 ^2^	0.715	0.227–1.522	2.05	0.021	151 (84.9%)	80.7%	92.1%
MSP1_42_ v274 ^2^	0.744	0.244–1.553	2.06	0.032	152 (85.5%)	79.8%	95.3
Non-immune subjects v88 ^3^	0.058	0.043–0.075	0.12	0.019	-		
NBSA ^1^					≥0.20		
infected patients:	0.752	0.375–1.344	3.38	0.062	161 (90.5%)	85.9%	98.4%
Non-immune subjects:	0.091	0.072–0.118	0.15	0.048	-		

A total of 178 samples were tested. NBSA, native blood stage antigen. ^1^ ABTS substrate and 60 min incubation. ^2^ TMB substrate and 20 min incubation with reads at 630 NM. *n*, number of samples; ^3^ v88 had higher OD values than other variants. No differences among recombinant proteins were observed: *X*^2^ = 1.48, d.f. = 2, *p* = 0.477.

## Data Availability

All relevant data are contained within the article or Supplementary Materials. Detailed data for this study are available under reasonable request.

## Supplementary Materials

The following supporting information can be downloaded at: https://www.mdpi.com/article/10.3390/life13030704/s1. Figure S1: ELISA IgG OD values and controls from six immuno-plates run for each PvMSP1_42_ protein/NBSA. Figure S2: Distribution of IgG OD values of samples tested by ELISA using PvMSP1_42_ recombinant proteins and the NBSA as coating antigens. Figure S3: Median-joining network using *pvmsp1_42_* sequences of parasites form southern Mexico and Nicaragua.
