# Supplementary material for: Plasmodium vivax MSP1-42 kD Variant Proteins Detected Naturally Induced IgG Antibodies in Patients Regardless of the Infecting Parasite Phenotype in Mesoamerica
Source: Life (Basel). 2023 Mar 6;13(3):704. doi: 10.3390/life13030704 (PMC10058798; doi:10.3390/life13030704)
Supplement: Supplementary file 1 [file life-13-00704-s001.zip › life-2222584-supplementary.pdf]

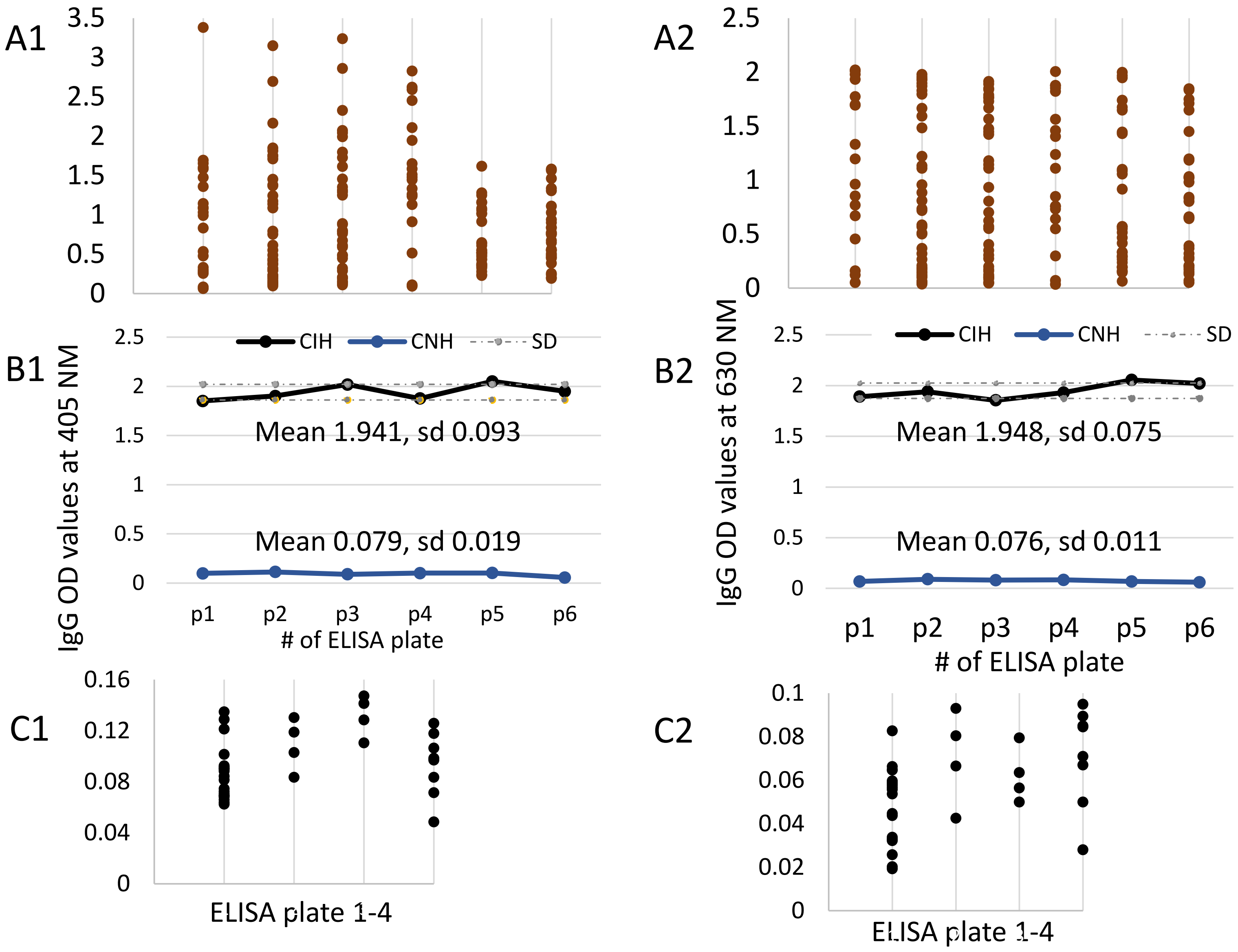

Figure S1. ELISA IgG OD values and controls from six immunoplates run for each PvMSP1$_{42}$ protein/NBSA. Graphs shows the OD values to NBSA (1) and to PvMSP1$_{42}$ v88 (2). A) indicate values of samples from *P. vivax* patients tested per plate (p1-p4 samples from southern Mexico and p5-p6 samples from Nicaragua); B) Controls: OD values by a pool of negative non-immune (CNH) and a pool of immune (CIH) plasma samples per plate (p1-p6); C) Nonimmune plasma samples (n=36) were distributed in the first four plates. All plates were run using the same batch of reagents and carried out by the same person.

Figure S2 Distribution of IgG OD values of samples tested by ELISA using $PvMSP1_{42}$ recombinant proteins or NBSA as coating antigens.
(a) ELISA test using NBSA was developed by using ABTS substrate and reads were recorded after 60 min of incubation. (b) ELISA using $PvMSP1_{42}$ e.g. v88 the reaction was developed by using TMB substrate and reads were recorded after 20 min of incubation before (1) and after (2) adding the stop solution.

(a)

(b1)

(b2)

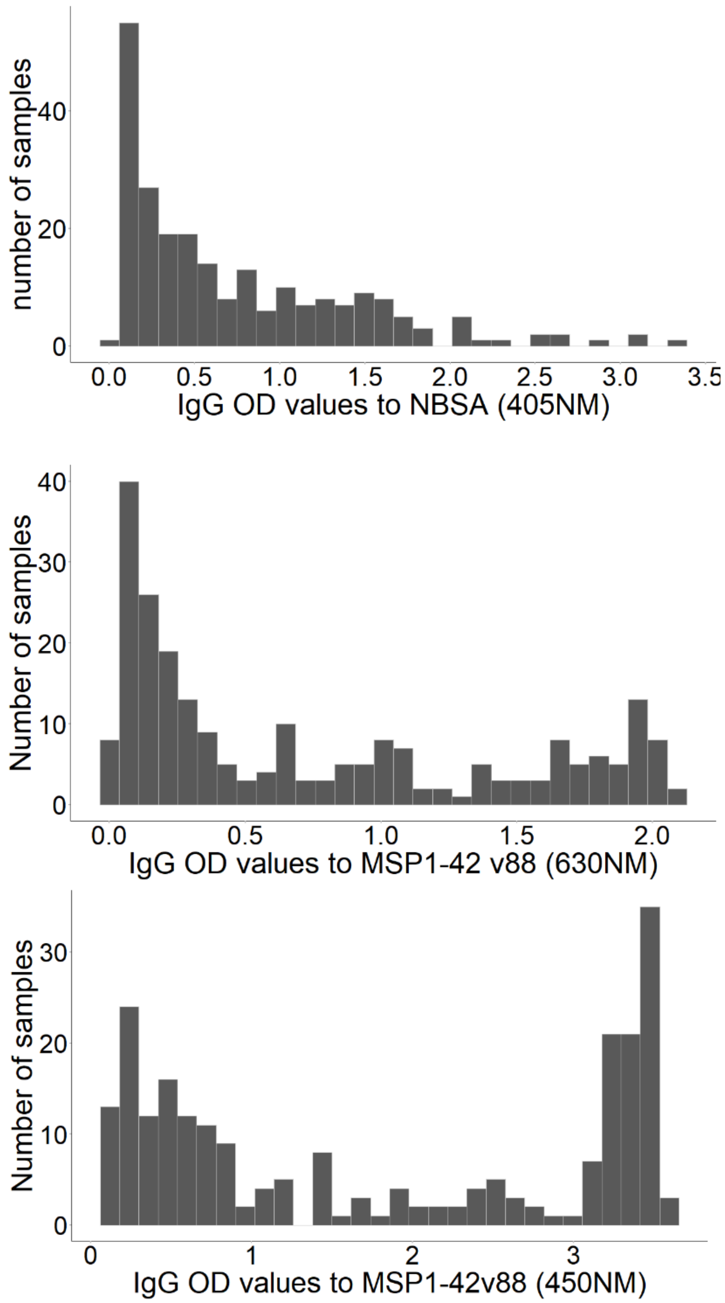

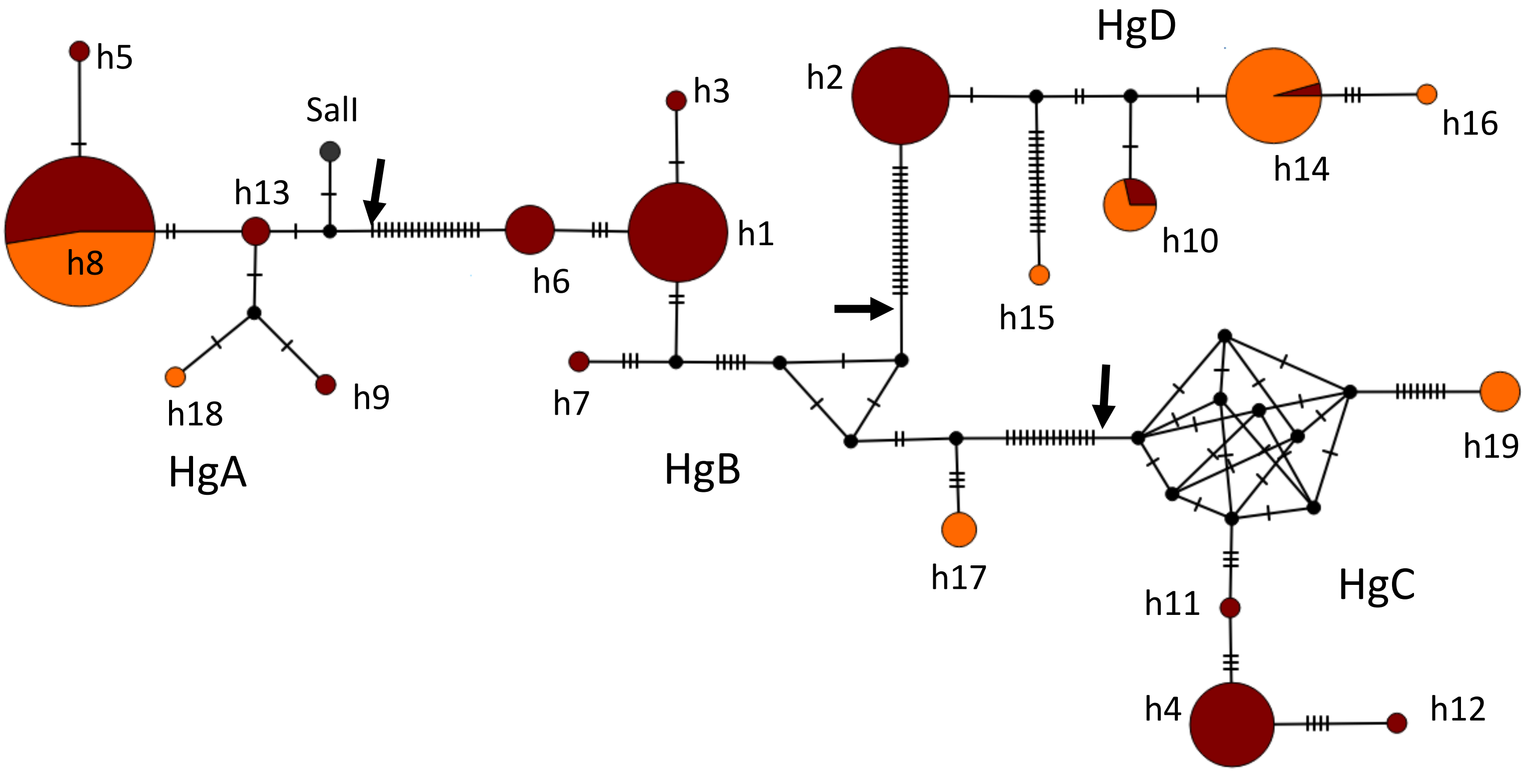

Figure S3. Median-joining network using *pvmsp1-42* sequences of parasites form southern Mexico (n=114; brown) and Nicaragua (n=64; orange). It shows 19 haplotypes and the Sal-I sequence. The haplotypes were grouped into four main haplogroups: A, B, C, and D. HgA: h8 (v88), h5, h9, h13, h18; HgB: h1 (v21), h6, h3, h7, h17; HgC: h4, h11, h12, h19; HgD:h2 (v274), h14, h10, h15, h16. The size of the circle is the number of haplotypes. Recombinant proteins haplotype v88, v21, v274. Blue rays separate haplogroups. The haplotype network was constructed using Population Analysis with Reticulate Trees (PopART) v1.7 [ref. 25]
